# Thiopurines Analogues with Additional Ring: Synthesis, Spectroscopic Properties, and Anticancer Potency

**DOI:** 10.3390/ijms24108990

**Published:** 2023-05-19

**Authors:** Katarzyna Krancewicz, Karolina Nowicka-Bauer, Katarzyna Fiedorowicz, Bronislaw Marciniak, Katarzyna Taras-Goslinska

**Affiliations:** 1 Faculty of Chemistry, Adam Mickiewicz University, Uniwersytetu Poznanskiego 8, 61-614 Poznan, Poland; katwol1@amu.edu.pl (K.K.); marcinia@amu.edu.pl (B.M.); 2 Centre for Advanced Technology, Adam Mickiewicz University, Uniwersytetu Poznanskiego 10, 61-614 Poznan, Poland; karolina.nowicka-bauer@amu.edu.pl; 3 Nanobiomedical Centre, Adam Mickiewicz University, Wszechnicy Piastowskiej 3, 61-614 Poznan, Poland; katfie1@amu.edu.pl

**Keywords:** tricyclic thiopurines, synthesis, physicochemical properties, anticancer potency, drug-likeness

## Abstract

Purine scaffolds constitute a starting point for the synthesis of numerous chemotherapeutics used in treating cancer, viruses, parasites, as well as bacterial and fungal infections. In this work, we synthesized a group of guanosine analogues containing an additional five-membered ring and a sulfur atom at the C-9 position. The spectral, photophysical, and biological properties of the synthesized compounds were investigated. The spectroscopic studies revealed that a combination of the thiocarbonyl chromophore and the tricyclic structure of guanine analogues shifts the absorption region above 350 nm, allowing for selective excitation when present in biological systems. Unfortunately, due to the low fluorescence quantum yield, this process cannot be used to monitor the presence of these compounds in cells. The synthesized compounds were evaluated for their effect on the viability of human cervical carcinoma (HeLa) and mouse fibroblast (NIH/3T3) cells. It was found that all of them display anticancer activity. In vitro studies were preceded by in silico ADME and PASS analyses, which confirmed that the designed compounds are promising candidates for anticancer agents.

## 1. Introduction

The purine ring is an important structural component of many naturally occurring N-heterocycles, such as nucleic acids, nucleoside metabolites, coenzymes, and alkaloids. Over recent years, the modified purine skeleton has become one of the most common elements used in drug design and development [1]. Due to their structural similarity to purines found in organisms, the new drugs based on purine scaffolds can be incorporated into metabolic pathways and hamper the synthesis of nucleic acids or inhibit the critical enzymes involved in the cellular metabolism of many viruses, parasites, bacteria, and cancers [2,3].

The thiopurines, such as 6-mercaptopurine or 6-thioguanine, are examples of drugs based on the purine ring system. They are used in the treatment of cancer, especially lymphoblastic leukemia in children [4,5] and are increasingly used in the treatment of Crohn’s disease [6]. The presence of a thiocarbonyl group shifts the absorption region of thiopurines towards longer wavelengths compared to naturally occurring nucleosides [7]. This enables them to be effectively photoactivated when present in biological systems, which, together with near unity triplet quantum yields, increases the interest in developing them for oncological applications [8].

The other well-known groups of purine-based drugs are acyclovir (ACV) and ganciclovir (GCV). These modified guanine analogues are used in the treatment of herpesvirus diseases, including cytomegalovirus and varicella-zoster infections [9,10]. In addition, subsequent modifications transforming them into tricyclic analogues (an additional ring was added to the guanine backbone) have yielded a very promising group of antiviral drugs. The tricyclic analogues of ACV and GCV often exhibited better selectivity, lower toxicity, and improved physicochemical properties as potential drugs than their parental compounds [11,12,13]. The interest in studies on the group of tricyclic purine analogues has increased in recent years due to their promising biological properties. Wen-Lian Wu et al. developed the new tricyclic scaffold into a very potent and selective series of DPP-4 (Dipeptidyl peptidase IV) inhibitors for the treatment of type 2 diabetes [14]. Etheno derivatives of 2-aminopurine, in turn, were shown to be effective inhibitors of the bacterial (*E. coli*) purine-nucleoside phosphorylase (PNP) [15].

**Figure 1:**
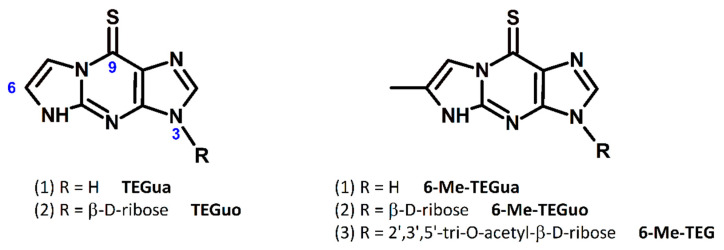
Structures of synthesized compounds.

Considering the improved physicochemical parameters and the higher selectivity and lower toxicity of the tricyclic purine analogues than the parent compounds, as well as the high activity of thiopurines as anticancer drugs, we decided to synthesize a new group of tricyclic thiopurine analogues (Figure 1). They were expected to exhibit comparable anticancer activity to the currently used thiopurine drugs but more favorable physicochemical parameters, which should result in less toxicity to healthy, untransformed cells. In the present work, we have undertaken studies on the synthesis and characterization of the photophysical properties of tricyclic thiopurines. A series of synthesized compounds were evaluated in silico and in vitro on HeLa cancer cells and mouse fibroblasts (NIH/3T3) to assess their potential as anticancer agents.

## 2. Results and Discussion

### 2.1. Chemistry and Spectral Characterization

Tricyclic thiopurine derivatives are a new group of compounds containing two biologically relevant chromophores, i.e., the tricyclic 1,N^2^-ethenoguanosine and the thiopurine framework. The compounds presented in this work were synthesized in a two-step procedure. First, a tricyclic linear structure was obtained by reacting guanosine with chloroacetaldehyde or bromoacetone. Depending on the type of reagent, tricyclic guanosine analogues with different substituents at the C-6 position were formed. Secondly, the thionation reaction of synthesized compounds afforded the final tricyclic thiopurine analogues: TEGuo (9-thio-1,N^2^-ethenoguanosine) and 6-Me-TEGuo (6-methyl-9-thio-1,N^2^-ethenoguanosine) (for structures, see Figure 1).

**Scheme 1:**
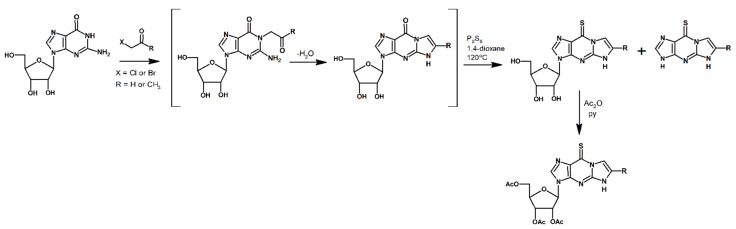
Reaction pathway for the synthesis of tricyclic thiopurine analogues.

During the second step of the synthetic procedure, the corresponding bases were formed as a minority product. After thionation, we received TEGua (9-thio-1,N^2^-ethenoguanine) and 6-Me-TEGua (6-methyl-9-thio-1,N^2^-ethenoguanine). In addition, the tricyclic thiopurine derivative 6-Me-TEGuo with blocked hydroxyl groups, designated 6-Me-TEG, was the compound we investigated as a model system in which our compounds would be cross-linked in a strand or appear as a nucleotide. The reaction pathway for the synthesis of tricyclic thiopurine analogues is presented in Scheme 1. The structures of all products were characterized by spectroscopic (^1^H NMR, ^13^C NMR) and mass spectrometric (HRMS (ESI)) methods. These data are given in the Experimental Section.

**Figure 2:**
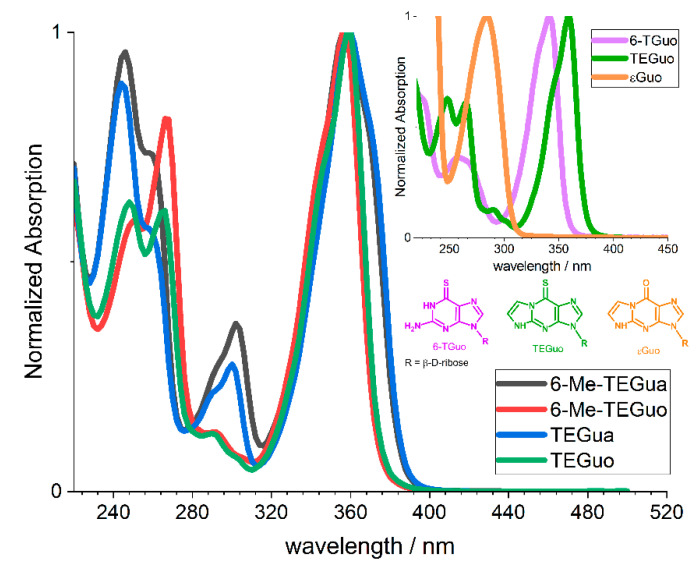
Normalized absorption spectra of the synthesized compounds in water. Inset: normalized absorption spectra of 6-thioguanosine (6-TGuo), 9-thio-1,N^2^-ethenoguanosine (TEGuo), and 1,N^2^-ethenoguanosine (εGuo) in water.

The new group of synthesized compounds was also characterized by UV-vis spectroscopy. The UV-vis spectra of all compounds in water are shown in Figure 2, whereas Table 1 presents spectral data including absorption maxima with corresponding absorption coefficients determined in protic and aprotic solvents, such as water (H_2_O), acetonitrile (ACN), ethanol (EtOH), ethylene glycol (EG), carbon tetrachloride (CCl_4_), and buffer solutions at pH 4.0 and pH 12.0. The choice of different solvents allowed us to observe the properties of the investigated compounds depending on the experimental conditions.

Tricyclic guanosine analogues show absorption in the UV region with a maximum of 280 nm (see inset in Figure 2). The substitution of a carbonyl oxygen atom for a sulfur atom in the tricyclic 1,N^2^-ethenoguanosine (εGuo) resulted in a shift of the absorption maximum to 360 nm in water (Figure 2) and a significant increase in the values of molar absorption coefficients (Table 1). Absorption in the 330–340 nm range is characteristic for 6-thioguanine, 6-thioguanosine, or 6-mercaptopurine. The thiocarbonyl chromophore found in the presented group of tricyclic thiopurine derivatives causes the maximum of the compounds studied to be around 360 nm.

The absorption properties of synthesized nucleosides and bases do not differ significantly. The acetyl groups in the nucleoside 6-Me-TEG do not affect the spectral properties of the nucleoside from which it derives.

The absorption spectra of tricyclic guanosine’s analogues with thiocarbonyl groups (TEGuo, 6-Me-TEGuo, TEGua, 6-Me-TEGua, and 6-Me-TEG) recorded in various solvents revealed a broad intensive band with a maximum in the range 350–365 nm depending on the solvent used. The position of this band and the large value of its molar extinction coefficient indicate that it is associated with the S_0_ → S_2_ electronic transition of π, π* character, as observed for other thiopurine analogues [16,17,18,19].

### 2.2. Tautomeric and Acid-Base Equilibria

The determination of a potential drug’s structure is important because any change in the form of a molecule can directly affect its therapeutic properties. The presence of a sulfur atom at the C-9 position in tricyclic thiopurine derivatives allows these compounds to exist in two different tautomeric forms: thione and thiol. For previously studied compounds, such as 6-thioguanine, 6-thioguanosine, or 6-mercaptopurine, the thione form was confirmed to dominate [20,21]. We have shown by ab initio calculations that the thione form also dominates over the thiol form in our previous work on a tricyclic thiopurine analogue with two thiocarbonyl groups at the C-9 and C-6 positions (DTEG) [16]. Comparing the literature reports on thiocarbonyl compounds and our published results for DTEG, we conclude that the currently investigated tricyclic thiopurine analogues also exist in the thione form. Furthermore, studies of various tautomeric forms of 1,N^2^-ethenoguanine (a guanine analogue with an additional five-membered ring) and its derivatives have shown that these compounds also contain a carbonyl rather than an OH group in their structure [15].

The synthesized compounds, due to their structure, can be hydrogen donors as well as acceptors. Depending on the pH, they can exist in protonated or deprotonated forms. In order to establish which form of the new tricyclic thiopurine derivatives is favored at neutral pH (the conditions under which the in vitro tests will be carried out), their pK_a_ values were determined by spectrophotometric methods (see Table 1 and the Experimental Section). The pK_a_ for TEGuo, 6-Me-TEGuo, and the corresponding bases were found to be 8.6 and 8.8, respectively. This means that at physiological pH (7.32–7.42), about 6% of TEGuo/TEGua exist in ionic form, while 6-Me-TEGuo/6-Me-TEGua only about 4%.

By analogy with the literature [15,16,20,21] and the results that we obtained, we conclude that the series of compounds in this work exist in the thione form at physiological pH.

### 2.3. Lipophilicity Studies

The key parameter in drug design and one of the most important properties for all the pharmacokinetic rules is the octanol/water partition coefficient, log P_o/w_ [22]. It is defined as the partition coefficient ratio of a compound between the hydrophobic and hydrophilic phases and corresponds to lipophilicity, which describes drug interactions with membranes and the possibility of their transport across the membrane into the cell. The values of log P_o/w_ were determined experimentally for all the tested compounds and compared with the values theoretically estimated on the SwissADME [23] webserver. The results are summarized in Table 2.

As seen in Table 2, temperature did not significantly affect the logP values for all the compounds analyzed. However, at 37 °C, the logP values were lower than at 25 °C. This may be due to the increased solubility of the compounds in the aqueous layer at the higher temperature. The partition coefficients for the synthesized compounds at both 25 and 37 °C increase in the order of TEGuo, 6-Me-TEGuo, TEGua, 6-Me-TEGua, and 6-Me-TEG, suggesting that the lipophilicity of these compounds also increases in the same way.

The predicted logP values for all the compounds analyzed reflect the same trend in the lipophilicity of the molecules studied. The observed differences in the values obtained with the two methods can be explained by the limitations of each method. Firstly, the methods implemented by SwissADME do not take into account the dissociation process that the molecules undergo under experimental conditions. Secondly, the concentration of compounds in the aqueous phase is measured spectrophotometrically. Therefore, the concentration obtained depends on the value of the molar extinction coefficient, which is used for concentration calculations. The calculation neglects tautomeric equilibrium and conformational changes or dimer formation that may occur in solution. However, despite the differences in logP values determined theoretically and experimentally, SwissADME predictions allow an estimate of the relative lipophilicity of the compounds studied.

### 2.4. ADME and PASS Analyses

In the search for new potential compounds with pharmaceutical applications, various methods are used to test the possibility of their use [24,25]. The initial step to considering a compound as a drug candidate is an ADME prediction. Both building blocks of tricyclic thiopurine analogues, i.e., etheno derivatives of purines and purines containing a thiocarbonyl group, have the potential to be used as drugs [4,11,14]. Therefore, we decided to perform an ADME analysis for the synthesized compounds to verify their pharmacological potential.

In the work presented here, the physicochemical properties and drug-likeness of a group of tricyclic thiopurine analogues were predicted using the SwissADME [23] webserver. The results can be found in Table 3 and Table 4.

The obtained parameters, such as molecular weight (MW), number of hydrogen bond donors (HBD), number of hydrogen bond acceptors (HBA), and octanol/water partition coefficient (Mlog P), were evaluated using the Lipinski rule of five (L-Ro5) [26]. All the studied molecules were acceptable under this criterion. Topological polar surface area (TPSA) and the number of rotational bonds (RB), which directly affect membrane permeability, were also calculated. Only the bases tested had RB values ≤ 10 and TPSA values less than 140 Å^2^. The lower TPSA and higher logP values for the bases made them more favorable than the nucleosides for passing through the intestinal membrane. This was reflected in high gastrointestinal (GI) absorption. Furthermore, the results showed that none of the compounds would be able to cross the blood–brain barrier (BBB). Both the nucleosides and the bases have TPSAs higher than 70 Å^2^ and are rich in hydrophilic substituents. This makes it difficult for them to enter the brain. The susceptibility of drug candidates to interact with P-glycoprotein (P-gp) transporters as well as with cytochrome P-450 (CYP) enzymes is another key parameter to predict the potential toxicity of compounds. None of the nucleosides tested showed affinity for the P-glycoprotein (P-gp) or (CYP) isoenzymes. The tested bases were also classified as non-substrate glycoprotein permeases. However, in silico calculations showed that they could exert an inhibitory effect on CYP1A2. A final important parameter affecting drug bioavailability is the water solubility of compounds. The more water-soluble a compound is, the lower the pharmaceutical dose in a smaller volume is required to achieve the desired effect or the required pharmacological response. Being weakly lipophilic (logP less than or close to 0), all the compounds were predicted to be very soluble or soluble. The calculated descriptors listed above have a direct impact on the Bioavailability Score (BS), a parameter that determines the potential of a drug candidate to be absorbed and utilized by the body. All the compounds analyzed had a BS = 0.55 and met the L-Ro5 criteria. They can therefore be considered molecules with good bioavailability.

**Table 3:** Physicochemical properties of the synthesized compounds.

	Physicochemical Properties							
	MW/Da	HBA	HBD	MlogP	RB	TPSA/Å ^2^	logS ^1^	Solubility Class ^2^
6-Me-TEGuo	337.35	6	4	−1.04	2	152.92	−1.03	Very soluble
6-Me-TEGua	205.24	2	2	0.27	0	93.86	−2.14	Soluble
TEGuo	323.33	6	4	−1.71	2	152.92	−0.68	Very soluble
TEGua	191.21	2	2	−0.06	0	93.86	−1.80	Very soluble
6-Me-TEG	463.46	9	1	−0.03	8	171.13	−2.12	Soluble

MW (molecular weight), HBA (hydrogen bond acceptor), HBD (hydrogen bond donor), MlogP [27,28] (P is defined as the ratio of the compound’s organic (oil) to aqueous phase concentrations), RB (rotational bonds), TPSA (topological polar surface area), and logS (water solubility) values of the compounds were calculated by SwissADME; ^1^ LogS in the table is the average value of logS calculated using three different methods derived by the SwissADME webserver; ^2^ Solubility class—logS scale: insoluble < −10 < poorly < −6 < moderately < −4 < soluble < −2 < very < 0 < highly.

**Table 4:** Pharmacokinetics and drug-likeness of the synthesized compounds.

	Pharmacokinetics				Drug-Likeness		Antineoplastic Activity
	BBB	GI	P-gp Substrate	CYP Inhibitor	BS	L-Ro5	Pa (Pi)
6-Me-TEGuo	No	Low	No	No	0.55	☑	0.744 (0.019)
6-Me-TEGua	No	High	No	No (except CYP1A2)	0.55	☑	0.728 (0.022)
TEGuo	No	Low	No	No	0.55	☑	0.775 (0.015)
TEGua	No	High	No	No (except CYP1A2)	0.55	☑	0.781 (0.014)
6-Me-TEG	No	Low	No	No	0.55	☑	0.819 (0.010)

BBB (blood–brain barrier permeant), GI (gastrointestinal absorption), P-gp (P-Glycoprotein) substrate, CYP (CYP1A2, CYP2C19, CYP2C9, CYP2D6, CYP3A4) inhibitor, and BS (Bioavailability Score) were determined by SwissADME; antineoplastic activity was determined by PASS.

The antineoplastic activity of the synthesized compounds was determined using an online version of PASS (Prediction of Activity Spectra for Substances) [29]. The Pa (probability of a compound to be active) and Pi (probability of a compound to be inactive) values of the designed and screened compounds are listed in Table 4. The results obtained showed that all the compounds tested showed Pa > 0.7 together with very low Pi values for antineoplastic activity; therefore, there is a very large chance to observe anticancer activity experimentally for those compounds.

### 2.5. Emission Properties

Modified nucleic acid bases often exhibit different physicochemical properties than naturally occurring nucleosides [7,30]. Some of them exhibit measurable fluorescence, making it possible to use them as fluorescent probes. If the compounds under discussion are characterized by high fluorescence quantum yields, they could provide a tool for observing and visualizing processes involving these structures in model and cancer cells. Although sulfur-modified pyrimidines or purines exhibit weak fluorescence from the S_2_ state with a yield of Φ_F_ ≤ 10^−3^ [7,31], some nucleobase analogues with an additional ring show fluorescence even with a yield of Φ_F_ = 0.6 [32]. In addition, the structural similarity of the synthesized compounds to wyosine, whose intrinsic fluorescence was observed in the anticodon loop in tRNA^Phe^ (Φ_F_ = 0.07 ± 0.01) and used for probing the conformational and binding properties of tRNA [33,34], prompted us to investigate the emission properties of the group of compounds that we studied.

**Figure 3:**
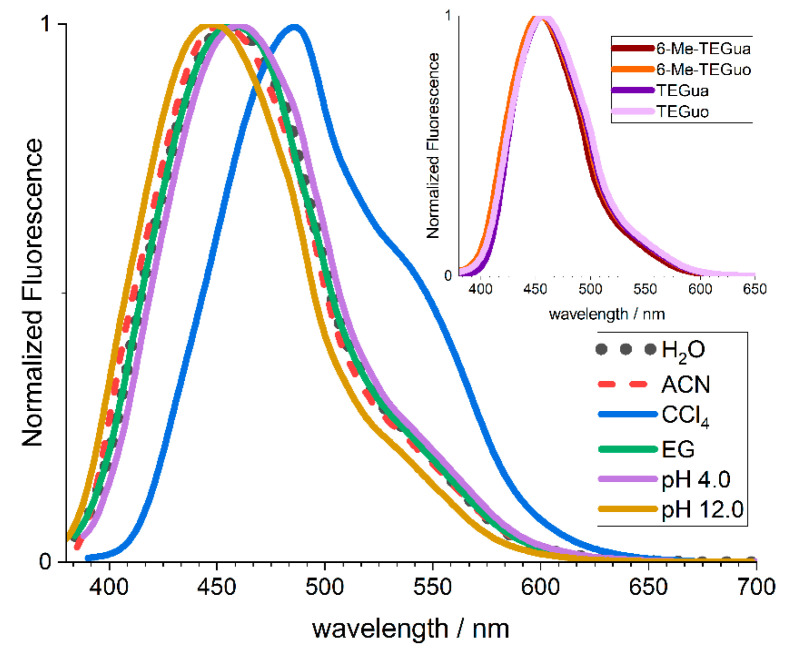
Normalized emission spectra of TEGuo in solvents of different polarities. Inset: normalized emission spectra of synthesized compounds in water.

Several solvents were proposed for the study, selected so that the effect of the environment on fluorescence intensity could be assessed. Environmental interactions with the emission of molecules are the subject of numerous works [35,36,37]. The reason for the solvent effect on fluorescence comes from non-covalent chemical interactions, both non-specific (e.g., London forces, dipole-dipole, dipole-induced dipole) and specific (hydrogen bond) in nature. The fluorescence properties of tricyclic thiopurine analogues were investigated in solvents of different polarities like H_2_O, EG, ACN, and CCl_4_, as well as in buffer solutions of pH 4.0 and pH 12.0 (Table 5). Under excitation at room temperature into the S_0_ → S_2_ absorption band (λ_exc_ = 360 nm), in air-equilibrated solutions, emission spectra with an intense band were observed with maxima in the range 440–490 nm, depending on the solvent polarity (Figure 3). The shape and position of the excitation spectra of the observed emission were consistent with the absorption spectra of the tested compounds. The emission bands were not quenched by molecular oxygen, suggesting that the emission bands were due to fluorescence. The lifetimes of fluorescence were determined using the Time-Correlated Single Photon Counting method with excitation at a 378 nm wavelength. Fluorescence kinetics exhibited monoexponential decay with lifetimes in the range of 9 to 16 ns, depending on the solvent used (Table 5). The fluorescence quantum yield (Φ_F_) values were determined following the relative method with 10-methylacridinium cation as a reference (Φ_F_ = 1.0 in water) [38]. The fluorescence intensity of the tricyclic thiopurine analogs was the highest for CCl_4_ solutions. Carbon tetrachloride, as an inert solvent, shows weak interactions with the dissolved molecules. Thus, the fluorescence quantum yield (Φ_F_~10^−2^) was increased in CCl_4_ compared to polar solvents. In polar solvents, such as water and ethylene glycol, the possibility of hydrogen bonding and non-specific interactions may affect the fluorescence properties of molecules. Ethylene glycol serves as a model system for studying complex intramolecular and intermolecular interactions in biologically significant compounds [39]. It is more viscous than water, imitating the cell environment, and is characterized by a less mobile three-dimensional hydrogen bonding network than in water. Therefore, both water and ethylene glycol were used to evaluate the impact of the hydrogen bonds on the fluorescent intensity of the tricyclic thiopurine analogues. It was found that the number of hydrogen bonding sites as well as the density of the solvent do not influence the fluorescent properties of the studied molecules. Moreover, in all polar solvents (a similar observation was reported for acetonitrile solution), the quantum yield of fluorescence was reduced to Φ_F_~10^−3^ compared to CCl_4_. This decreasing quantum efficiency may be related to both non-specific solvent interactions and specific solute-solvent interactions, including hydrogen bonding.

The fluorescence emission of the studied compounds was found to be dependent on pH. At pH 4.0, as was the case for the absorbance at 352 nm, the intensity and shape of the fluorescence signal were similar to those observed in water solution for all the compounds tested. However, in the case of basic pH, a two-fold increase in the emission intensity and a blueshift of the emission maximum were observed. The pK_a_* determined, taking into account the emission from the singlet excited state, was estimated to be 8.7 and 8.9 for TEGuo and 6-Me-TEGuo, respectively.

The low quantum yield of the emission, Φ~10^−3^, the small effect of the environment on the fluorescence intensity, and the short fluorescence lifetime unfortunately preclude the use of emission as a detection method for these compounds in biological systems.

### 2.6. Biological Evaluation: Influence on Cell Viability

The compounds under investigation have been designed as potential anticancer agents. These drugs are expected to exhibit high activity and selectivity with low toxicity to healthy cells. The problem with current anticancer drugs is the negative side effects that accompany therapies using them. Currently, new drugs with more favorable properties are being sought that simultaneously reduce the negative effects of their use. The analyzed compounds are a combination of components that exhibit pharmacological activity. The first component is the thiopurine chromophore. Thiopurines are used in anti-cancer and anti-viral therapies [4,40] and in transplantology [41]. However, long-term therapy with thiopurines can cause adverse effects at different levels: myelosuppression, hepatotoxicity, pancreatitis, and gastrointestinal intolerance [42].

The second building block is an additional ring present in the structure of etheno derivatives of purines. Tricyclic guanine analogues are compounds widely reported in the literature with known biological applications; e.g., such modifications of acyclovir can be used in antiviral therapies [12]. The combination of these two types of compounds offers the possibility of obtaining new compounds with high anticancer activity while reducing their toxicity to healthy cells.

**Figure 4:**
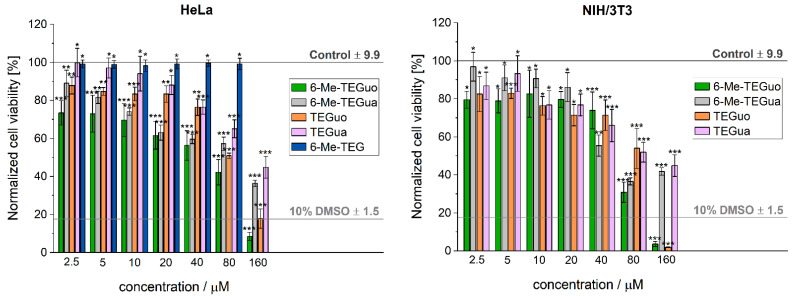
Effect of the compounds tested (in various concentration ranges) on the viability of HeLa and NIH/3T3 cells after 24 h of incubation. Data presented as a mean ± SD (*n* = 4); * *p* < 0.05, ** *p* < 0.01, *** *p* < 0.001—significant difference compared to the control. The data were normalized against control (not-treated) cells.

**Figure 5:**
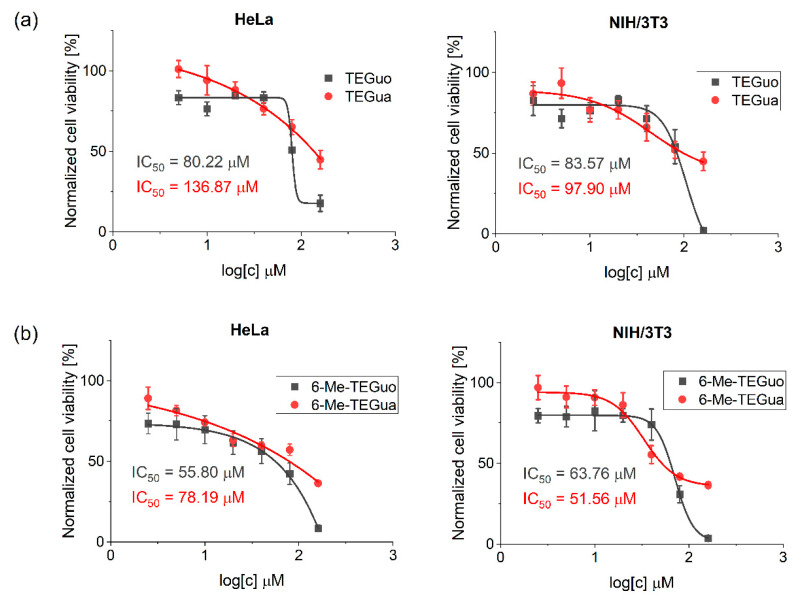
Half maximal inhibitory concentration (IC_50_) of TEGuo and TEGua (**a**) and 6-Me-TEGuo and 6-Me-TEGua (**b**) in HeLa and NIH/3T3 cells. Cells were treated with increasing concentrations of compounds for 24 h. Cell viability was assessed by the CellTiter-Glo^®^ 2.0 assay. The data are expressed as mean ± SD (*n* = 4), and IC_50_ values are indicated. The data were normalized against control (not-treated) cells.

The compounds in this work were tested for their in vitro inhibitory effect on the viability of the HeLa (cancerous) cell line using the CellTiter-Glo^®^ 2.0 cell viability assay (24 h). In addition, the mouse fibroblast NIH/3T3 cell line was used to assess the effects of the compounds on healthy normal cells. The cells were treated with varying concentrations of the synthesized compounds, ranging from 2.5 μM to 180 μM (the maximum concentration was limited by the solubility of the tested compounds). The 6TG was used as a standard in all tests conducted. The graph of percentage cell viability (y axis) against concentrations of the tested compounds (x axis) and concentration-viability curves with IC_50_ values from dose-response studies are presented in Figure 4 and Figure 5, respectively.

It was observed that all the new compounds affected cell viability in a dose-dependent manner. The best activity among the analyzed compounds for HeLa cells was shown by 6-Me-TEGuo (IC_50_ = 55.80 μM), 6-Me-TEGua (IC_50_ = 78.19 μM), and TEGuo (IC_50_ = 80.22 μM). Evaluation of the effects of the analyzed compounds on normal NIH/3T3 cells indicated that all the compounds reduced cell viability in a dose-dependent manner. The following compounds had the lowest effect on NIH/3T3 cell viability: TEGua (IC_50_ = 97.90 μM) and TEGuo (IC_50_ = 83.57 μM).

**Figure 6:**
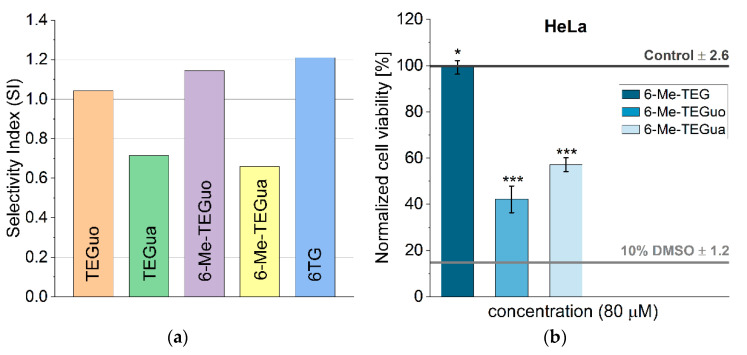
(**a**) Comparison of selectivity index (SI) values of the compounds tested; (**b**) Viability of HeLa cells treated with 6-Me-TEG, 6-Me-TEGuo, and 6-Me-TEGua (c = 80 μM) for 24 h as determined by the CellTiter-Glo^®^ 2.0 Assay. Data are presented as a mean ± SD (*n* = 4); * *p* < 0.05, *** *p* < 0.001—significant difference compared to the control. The data were normalized against control (not-treated) cells.

From the data presented, it appears that the synthesized nucleosides (TEGuo and 6-Me-TEGuo) have an SI > 1, as does 6TG, which is already being used to treat patients. The selectivity index (SI) was calculated using the formula: SI = (IC_50_ normal cell (NIH/3T3))/(IC_50_ cancerous cell (HeLa)). The calculated SI values for the novel compounds and 6TG are presented in Figure 6a.

The investigated compounds become potential candidates for further, more detailed studies. For the etheno analogues of guanosine, which were synthesized in the first step of the synthetic procedure of tricyclic thiopurine analogues (see Experimental Section), it was not possible to determine the IC_50_ under the conditions under which our sulfur derivatives were tested. The literature also reports that ethenoguanosine derivatives do not show cytotoxicity on tumor cells, including HeLa [43].

In our experiments, the substitution of an oxygen atom for a sulfur atom in the tricyclic purine derivatives results in a definite increase in their interaction with cancer cells.

It is known that tricyclic purine derivatives can be formed in living organisms as a result of the action of harmful agents, e.g., aldehydes [44]. The occurrence of such structures, where nucleic acid bases have an extra 5-membered ring attached, was confirmed and observed, e.g., in modified DNA/RNA strands [45]. By analogy with the reactions that purines undergo, there is a large probability for the formation of such tricyclic thiopurine derivatives in biological systems. In particular, patients exposed to therapies using thiopurines that are incorporated into nucleic acid structures may be at risk for the presence of these compounds. Therefore, in studies on the HeLa cell line, we tested the compound 6-Me-TEG, with blocked OH groups, as a model for tricyclic derivatives embedded in the RNA/DNA strand.

6-Me-TEG was tested under the same conditions as the other thio analogs for activity against HeLa cells. In the concentration range from 2.5 μM to 80 μM, no effect on HeLa cell viability was observed (Figure 6b). In comparison, the free nucleoside (6-Me-TEGuo) at the same concentration reduced cell viability by up to 42%, while the free base (6-Me-TEGua) reduced cell viability by up to 57%.

## 3. Materials and Methods

### 3.1. Chemicals

All reagents and solvents were obtained from Sigma-Aldrich Chemical Co. (St. Louis, MO, USA), Merck (Darmstadt, Germany), and TriMen Chemicals (Łódź, Poland). Water was purified on a Milli-Q system (Millipore, Bedford, MA, USA). 6-thioguanine (6TG) used in the in vitro studies was purchased from Merck.

### 3.2. Identification Methods

The course of reactions was monitored by thin layer chromatography (TLC) using aluminum Merck TLC plates coated with silica gel 60 F254. The ^1^H and ^13^C NMR spectra were measured in a MeOD solution at 600 MHz (^1^H) and 151 MHz (^13^C) using a BRUKER ASCEND™ 600 MHZ spectrometer. Chemical shifts were reported in parts per million (ppm) with reference to the residue solvent peak for ^1^H and ^13^C (^1^H δH = 3.36 and 4.94 ppm, ^13^C δC = 47.66 ppm for MeOD). Mass spectra were obtained with HRMS (ESI) analysis using an Impact HD QTOF Mass Spectrometer (BRUKER) equipped with a Thermo Scientific/Dionex UHPLC (UltiMate 3000).

### 3.3. Synthesis of Tricyclic Thiopurine Analogues

General Procedure A [46]: Guanosine was dissolved in anhydrous DMSO, and NaH was added. When the solution became clear, the ketone was dropped and stirred for 2 h at room temperature. Then, 0.5 N KOH was poured into the mixture and stirred overnight. The reaction was quenched by neutralizing it with acetic acid, which evaporated to dryness. The crude mixture was purified by the 971-FP Flash Purification System from Agilent (Kinesis TELOS Flash Chromatography Column, Silica, 12 g). A gradient of methanol in ethyl acetate was used as an eluent. The fractions containing the products were pooled, evaporated to dryness, and recrystallized twice from water.

General Procedure B [47]: Guanosine was dissolved in water, and aldehyde was added. The mixture was stirred at 37 °C for 4 days, and the pH was maintained between 6.35 and 6.45 using a 0.2 M aqueous sodium hydroxide solution. The reaction mixture was evaporated to dryness and purified by the 971-FP Flash Purification System from Agilent (Kinesis TELOS Flash Chromatography Column, Silica, 12 g). A gradient of methanol in ethyl acetate was used as an eluent. The fractions containing the products were pooled, evaporated to dryness, and recrystallized twice from water.

General Procedure C [48]: A mixture of the final product obtained from procedures A or B and P_2_S_5_ was dissolved in 1,4-dioxane and stirred at 120 °C for 3 h. Then, the solution was cooled to room temperature, and a saturated solution of NaHCO_3_ was added. The side products were extracted with chloroform, and the water fractions containing the products were evaporated to dryness. The main products were initially purified by the 971-FP Flash Purification System from Agilent (Kinesis TELOS Flash Chromatography Column, Silica, 12 g). A gradient of methanol in chloroform was used as an eluent. The fractions containing the products were pooled, evaporated to dryness, and purified again by a preparative HPLC system (Agilent 1260 Infinity HPLC System with DAD), equipped with a ZORBAX SB-C18 (150 mm × 9.4 mm i.d., 5 µm) column from Agilent (flow rate 5 mL/min). A gradient of acetonitrile in water was used as an eluent. Fractions containing products were collected and evaporated to dryness.

General Procedure D: The tricyclic thioguanosine analogue was dissolved in anhydrous pyridine, and acetic anhydride was added. After 3 h of stirring at room temperature in anhydrous conditions, when the reaction was finished, the solution was evaporated to dryness in vacuo several times with 2-propanol to remove the pyridine. The product was purified by a preparative HPLC system (Agilent 1260 Infinity HPLC System with DAD), equipped with a ZORBAX SB-C18 (150 mm × 9.4 mm i.d., 5 µm) column from Agilent (flow rate 5 mL/min). A gradient of acetonitrile in water was used as an eluent.

*6-methyl-9-thio-1,N^2^-ethenoguanosine (6-Me-TEGuo)/6-methyl-9-thio-1,N^2^-ethenoguanine (6-Me-TEGua)*: The title compounds were synthesized following, in the first step, General Procedure A using guanosine (2.0 g, 7 mmol), NaH (0.17 g, 7 mmol), bromoacetone (0.1 g, 7 mmol), DMSO (30 mL), and 0.5 N KOH (100 mL). 6-methyl-1,N^2^-ethenoguanosine was obtained as a main product (yield 75%); HRMS (ESI) calculated for C_13_H_15_N_5_O_5_ [M+H]+ 322.1151, found 322.1153. In the second step, General Procedure C was applied using 6-methyl-1,N^2^-ethenoguanosine (1.0 g, 3 mmol), P_2_S_5_ (2.01 g, 9 mmol), and 1,4-dioxane (20 mL). 6-methyl-9-thio-1,N^2^-ethenoguanosine (yield 53%) and 6-methyl-9-thio-1,N^2^-ethenoguanine (yield 35%) were obtained as the main products. 

*6-methyl-9-thio-1,N^2^-ethenoguanosine: ^1^H NMR (600 MHz, MeOD) δ (ppm)*: 1.92 (s, 1H, N5-H), 2.45 (s, 3H, -CH_3_), 3.80 (d, *J* = 3.4 Hz, 1H, C5′-H), 3.91 (d, *J* = 3.0 Hz, 1H, C5′-H), 4.15 (q, 1H, C4′-H), 4.37 (m, 1H, C3′-H), 4.74 (m, 1H, C2′-H), 6.03 (d, *J* = 5.6 Hz, 1H, C1′-H), 8.01 (s, 1H, C7-H), 8.36 (s, 1H, C2-H); ^13^C NMR (151 MHz, MeOD) δ (ppm): 9.48 (-CH_3_), 62.82 (C-5′), 69.15 (C-3′), 73.93 (C-2′), 85.89 (C-4′), 88.62 (C-1′), 107.33 (C-7), 128.72 (C-6), 130.13 (C-10), 140.77 (C-2), 143.24 (C-11), 144.14 (C-12), 166.13 (C=S); HRMS (ESI) calculated for C_13_H_15_N_5_O_4_S [M+H]^+^ 338.0923, found 338.0921

*6-methyl-9-thio-1,N^2^-ethenoguanine: ^1^H NMR (600 MHz, MeOD) δ (ppm):* 1.91 (s, 1H, N5-H), 2.44 (s, 3H, -CH_3_), 7.91 (s, 1H, C7-H), 8.57 (s, 1H, C2-H); HRMS (ESI) calculated for C_8_H_6_N_5_S [M+Na]^+^ 228.0319, found 228.0314 

*9-thio-1,N^2^-ethenoguanosine (TEGuo)/9-thio-1,N^2^-ethenoguanine (TEGua):* The title compounds were synthesized following, in the first step, General Procedure B using guanosine (1.0 g, 3.5 mmol), sodium chloride (3.53 g, 60 mmol), and chloroacetaldehyde (3.69g, 47 mmol) dissolved in 900 mL of water. 1,N^2^-ethenoguanosine was obtained as a main product (yield 45%); HRMS (ESI) calculated for C_12_H_13_N_5_O_5_ [M+H]+ 308.0994, found 308.0991. In the second step, General Procedure C was applied using 1,N^2^-ethenoguanosine (0.4 g, 1.3 mmol), P_2_S_5_ (0.87 g, 3.9 mmol), and 1,4-dioxane (10 mL). 9-thio-1,N^2^-ethenoguanosine (yield 56%) and 9-thio-1,N^2^-ethenoguanine (yield 32%) were obtained as the main products. 

*9-thio-1,N^2^-ethenoguanosine: ^1^H NMR (600 MHz, MeOD) δ (ppm)*: 1.92 (s, 1H, N5-H), 3.80 (d, *J* = 3.4 Hz, 1H, C5′-H), 3.91 (d, *J* = 3.0 Hz, 1H, C5′-H), 4.15 (q, 1H, C4′-H), 4.37 (m, 1H, C3′-H), 4.74 (m, 1H, C2′-H), 6.03 (d, *J* = 5.6 Hz, 1H, C1′-H), 7.53 (d, *J* = 2.4 Hz, 1H, C7-H), 8.26 (d, *J* = 2.4 Hz, 1H, C6-H), 8.36 (s, 1H, C2-H); ^13^C NMR (151 MHz, MeOD) δ (ppm): 61.53 (C-5′), 70.69 (C-3′), 74.16 (C-2′), 85.73 (C-4′), 88.62 (C-1′), 110.57 (C-6), 117.51 (C-7), 129.98 (C-10), 141.01 (C-2), 143.57 (C-11), 144.79 (C-12), 166.42 (C=S); HRMS (ESI) calculated for C_12_H_13_N_5_O_4_S [M+H]^+^ 324.0766, found 324.0764 

*9-thio-1,N^2^-ethenoguanine: ^1^H NMR (600 MHz, MeOD) δ (ppm)*: 1.91 (s, 1H, N5-H), 7.44 (d, *J* = 1.9 Hz, 1H, C7-H), 8.13 (d, *J* = 1.8 Hz, 1H, C6-H), 8.57 (s, 1H, C2-H); HRMS (ESI) calculated for C_7_H_5_N_5_S [M+H]^+^ 192.0343, found 192.0338 

*2′,3′,5′-tri-O-acetyl-6-methyl-9-thio-1,N^2^-ethenoguanosine (6-Me-TEG)*: The title compound was synthesized following General Procedure D using 6-methyl-9-thio-1,N^2^-ethenoguanosine (0.25 g, 0.7 mmol), anhydrous pyridine (20 mL), and acetic anhydride (2.5 mL). Yield 95%; ^1^H NMR (600 MHz, MeOD) δ (ppm): 2.06 (s, 3H, -CH_3_), 2.09 (s, 3H, -CH_3_), 2.17 (s, 3H, -CH_3_), 2.45 (s, 3H, C6-CH_3_), 4.44–4.39 (m, 2H, C5′-H), 4.50–4.45 (m, 1H, C4′-H), 5.74 (t, 1H, C3′-H), 6.09 (q, *J* = 5.3 Hz, 1H, C2′-H), 6.22 (d, *J* = 4.9 Hz, 1H, C1′-H), 8.01 (q, *J* = 1.2 Hz, 1H, C7-H), 8.22 (s, 1H, C2-H); ^13^C NMR (151 MHz, MeOD) δ (ppm): 9.48 (-CH_3_), 18.88 (-CH_3_), 19.05 (-CH_3_), 19.20 (-CH_3_), 62.82 (C-5′), 70.58 (C-4′), 72.56 (C-3′), 79.96 (C-2′), 86.85 (C-1′), 107.33 (C-7), 128.72 (C-6), 130.13 (C-10), 140.77 (C-2), 143.24 (C-11), 144.14 (C-12), 166.13 (C=S), 169.77 (5′-OC(O)-), 170.03 (3′-OC(O)-), 170.83(2′-OC(O)-); HRMS (ESI) calculated for C_19_H_21_N_5_O_7_S [M+H]^+^ 464.1239, found 464.1245.

The spectral data for the synthesized compounds are provided in Supporting Information (Figures S1–S8).

### 3.4. Photophysical Measurements

UV-vis absorption spectra were recorded using a Cary 100 spectrophotometer scanning from 800 to 200 nm with 1 nm increments in 1 cm × 1 cm quartz cells. Emission spectra were taken in 1 cm × 1 cm quartz cells on a JASCO FP–8300 spectrofluorometer (excitation and emission slits 5 nm, scan speed 500 nm min^−1^) for solutions with absorbance at the excitation wavelength less than 0.1. The fluorescence quantum yields were determined using 10-methylacridinium cation in water (Φ = 1.0) as a standard [38]. The dissociation constants were determined by the spectrophotometric method: in a first step, buffer solutions of different pH were prepared. The pH of solutions was determined by a Mettler Toledo F20 FiveEasy pH meter equipped with a pH electrode LE438 3 in 1 (Mettler Toledo). In the second step, equal volumes of a substance dissolved in water (10 μL) were added to the buffer solution (3 mL). The spectra were recorded with 1 nm resolution over the range from 220 to 550 nm to obtain different spectra around the maximum λ for each compound studied. The purity of all samples was monitored by an HPLC method. HPLC measurements were performed using an Agilent 1200 Rapid Resolution UHPLC system equipped with a reversed-phase column (Eclipse Plus C18, 5 mm, 4.6 × 250 mm). A gradient of acetonitrile in water was used as an eluent. The water was doubly distilled and purified using a Milli-Q system (Millipore, Bedford, MA, USA). The fluorescence lifetime measurements were recorded using a Fluorescence Lifetime Spectrometer (FluoTime 300 from PicoQuant) with a time-correlated single-photon counting detection system equipped with a 378 nm laser as its excitation source. All experiments were carried out in a standard quartz cell (1 cm × 1 cm).

### 3.5. ADME and PASS Analyses

Theoretical determination of the drug-like physicochemical and pharmacokinetic properties of the synthesized compounds was assessed using the SwissADME webserver (http://www.swissadme.ch, accessed on 20 November 2021) [23]. The compound’s 2D structural models were converted into SMILES using SwissADME’s SMILES generator and then analyzed to determine the compound’s ADME properties in terms of drug-likeness and pharmacokinetics. The antineoplastic activity of synthesized compounds was predicted using free Online version 2.0 of PASS (Prediction of Activity Spectra for Substances) [29]. The predicted activity spectrum was estimated and quantitatively expressed as the probability of a compound being active (Pa) and inactive (Pi).

### 3.6. Lipophilicity Determination

The partition coefficients of the tested compounds were determined for n-octanol/phosphate buffer pH 7.4 at 25 °C and 37 °C. The initial concentrations of the compounds (1.0 × 10^−5^ M and 2.0 × 10^−5^ M) were measured spectrophotometrically, according to the Beer–Lambert law. The absorbances were recorded at λ_max_ of the appropriate compound against a suitable phosphate buffer blank. A quantity of 1 mL of the solution of the tested compound in the phosphate buffer at pH 7.4 and 1 mL of n-octanol were placed in 2 mL Eppendorf tubes. The samples were shaken manually (2 min), vortexed (5 min), and incubated for 5 h at 25 °C or 37 °C. Then, the tubes were centrifuged (2 min, 10,000 rpm). Both layers were carefully separated, and the absorbances were measured for the aqueous layers at a suitable λ_max_ against the blank of the phosphate buffer at a suitable pH. The given logP_o/w_ values are the mean of three independent experiments (one experiment: 3 samples for 2 concentrations of each compound).

### 3.7. Cell Cultures and Toxicity Assays

The cytotoxicity of the synthesized compounds was tested using cell lines of cervical cancer (HeLa) and mouse fibroblasts (NIH/3T3). Both cell lines were cultured in Dulbecco′s Modified Eagle′s medium (31053, Sigma-Aldrich-Gibco, Thermo Fisher Scientific, Waltham, MA, USA) supplemented with 10% fetal bovine serum (Biowest, Nuaillé, France), 1% Glutamax (Thermo Fisher Scientific, Waltham, MA, USA), and 1% Antibiotic-Antimycotic (Biowest) under standard culture conditions (95% humidity, 5% CO_2_, at 37 °C). For the purpose of cytotoxicity assays, cells (from both cell lines) were seeded at a density of ca. 5000 cells/well on a 96-well microplate (white, flat bottom), in 4 replicates. 6-Me-TEGuo, 6-Me-TEGua, TEGuo, TEGua, and 6-Me-TEG were tested for their potential cytotoxicity using the following concentrations: 160 (except 6-Me-TEG), 80, 40, 20, 10, 5, and 2.5 μM. Additionally, 6-thioguanine (6TG) in concentrations of 10, 7.5, 5, 2.5, 1.25, and 0.625 μM was tested. All assays included non-treated cells and cells treated with 10% DMSO as negative and positive controls, respectively. The cells were incubated for 24 h under standard culture conditions. Cytotoxicity was measured as cell viability with the CellTiter-Glo^®^ Luminescent Cell Viability Assay (Promega, Madison, WI, USA) according to the manufacturer’s protocol. The microplate luminescence was measured using an Infinite^®^ 200 PRO plate reader (Tecan, Männedorf, Switzerland). Then, the average luminescence of non-labeled cells was equated to 100%, and the remaining values were proportionally normalized. The IC_50_ values were calculated from the fitting curves using Origin 2022.

### 3.8. Statistical Analysis

The statistical analysis of the data was performed using GraphPad Prism version 9.0.0 for Windows. Data were analyzed by one-way analysis of variance (ANOVA) followed by Tukey’s test (*p* < 0.05 vs. control). The results were expressed as the mean ± standard deviation (SD) and considered significant at a *p*-value ≤ 0.05.

## 4. Conclusions

Compared to thiopurines and analogues of guanine with an additional five-membered ring, the combination of the thiopurine chromophore with the tricyclic structure of guanine resulted in a shift of the absorption region towards longer wavelengths. The location of the absorption bands in the λ > 350 nm region, in contrast to the absorbance of nucleic acid and protein components, allows selective excitation of our compounds in biological systems. Their low fluorescence quantum yield, Φ_F_~10^−3^, suggests that the singlet excited state of the tricyclic thiopurine analogs decays mainly by non-radiative processes. The presented group of tricyclic thiopurine derivatives behaves like typical thiocarbonyl compounds, and the processes that take place in their excited singlet states are not very efficient. The characterization of the triplet excited states of these synthesized compounds will answer the question of whether the processes occurring in these states can be used in biological research.

In silico prediction of ADME properties, toxicity, and drug-likeness suggests that all the compounds are promising candidates for the development of biologically active agents. In vitro analysis showed that TEGuo, 6-Me-TEGuo, and 6-Me-TEGua exhibited antitumor activity against HeLa cells with an IC_50_ < 100 μM. However, studies in more selected cell lines, e.g., acute lymphoblastic leukemia (ALL), acute myeloid leukemia (AML), and colon cancer (LoVo), will allow us to determine the potential applications of this new group of compounds.

## Figures and Tables

**Table 1:** Absorption properties of synthesized compounds in different solvents.

	ACN	H_2_O ^1^	CCl_4_^2^	pH = 4.0	pH = 12.0	pK_a_
λ_Abs max/_nm (ε_max/_M^−1^ cm^−1^)						
TEGua	353 (21,200)	350 (21,800)	364	352 (21,200)	362 (13,300)	8.6
TEGuo	355 (23,400)	352 (23,900)	362	352 (23,300)	363 (14,600)	8.6
6-Me-TEGua	353 (21,300)	350 (22,000)	364	353 (21,500)	360 (13,600)	8.8
6-Me-TEGuo	355 (23,400)	352 (24,000)	361	352 (23,400)	359 (14,800)	8.8

^1^ the position of the absorption maximum was the same for ethanol and ethylene glycol solutions; ^2^ the molar extinction coefficients were not able to be determined due to the limited solubility of compounds in CCl_4._

**Table 2:** Experimentally determined log P_o/w_ values for 1-octanol/phosphate buffer solution measured at pH 7.4 at 25 °C and 37 °C and theoretically estimated consensus log P_o/w_ values for synthesized compounds.

	Experimental log P_o/w_ (pH 7.4)		Calculated log P_o/w_
	25 °C	37 °C	Consensus log P_o/w_
6-Me-TEGuo	−0.61	−0.67	−0.44
6-Me-TEGua	0.75	0.70	1.14
TEGuo	−0.89	−0.96	−0.80
TEGua	0.58	0.52	0.81
6-Me-TEG	0.80	0.75	0.93

**Table 5:** Emission properties of synthesized compounds in different solvents.

	λ _max/_nm	Φ_F_	τ/ns	Solvent
TEGua	450	0.004	14.6	ACN
	457	0.004	15.8	H_2_O/EG ^1^
	486	0.012	12.6	CCl_4_
TEGuo	451	0.005	14.0	ACN
	457	0.005	16.1	H_2_O/EG ^1^
	487	0.014	16.6	CCl_4_
	460	0.004	14.1	pH = 4.0
	448	0.011	18.5	pH = 12.0
6-Me-TEGua	440	0.006	14.0	ACN
	454	0.007	11.6	H_2_O/EG ^1^
	484	0.012	11.6	CCl_4_
6-Me-TEGuo	440	0.006	14.7	ACN
	455	0.006	9.4	H_2_O/EG ^1^
	486	0.013	10.8	CCl_4_
	459	0.005	11.5	pH = 4.0
	445	0.012	13.5	pH = 12.0

^1^ the values of maximum fluorescence and fluorescence quantum yields in ethylene glycol were the same as in water. The lifetimes of emissions in ethylene glycol were not determined.

## Data Availability

All data generated or analyzed during this study are included in this published article.

## Supplementary Materials

The following supporting information can be downloaded at: https://www.mdpi.com/article/10.3390/ijms24108990/s1.
