# Supplementary material for: Thiopurines Analogues with Additional Ring: Synthesis, Spectroscopic Properties, and Anticancer Potency
Source: Int J Mol Sci. 2023 May 19;24(10):8990. doi: 10.3390/ijms24108990 (PMC10219169; doi:10.3390/ijms24108990)
Supplement: Supplementary file 1 [file ijms-24-08990-s001.zip › ijms-2383839-supplementary.pdf]

Electronic Supporting Information

Thiopurines analogues with additional ring: synthesis, spectroscopic properties and anticancer potency

Katarzyna Krancewicz [1], Karolina Nowicka-Bauer [2], Katarzyna Fiedorowicz [3], Bronislaw Marciniak [1,2] and Katarzyna Taras-Goslinska [1,*]

[1] Faculty of Chemistry, Adam Mickiewicz University, Uniwersytetu Poznanskiego 8, 61-614 Poznan, Poland
[2] Centre for Advanced Technology, Adam Mickiewicz University, Uniwersytetu Poznanskiego 10, 61-614 Poznan, Poland
[3] Nanobiomedical Centre, Adam Mickiewicz University, Wszechnicy Piastowskiej 3, 61-614 Poznan, Poland

* Correspondence: karem@amu.edu.pl

^{1}H NMR and ^{13}C NMR of compounds

Content:

1. ^{1}H and ^{13}C NMR spectra of TEGuo (Figs S1–S2)
2. ^{1}H spectrum of TEGua (Fig. S3)
3. ^{1}H and ^{13}C NMR spectra of 6-Me-TEGuo (Figs S4–S5)
4. ^{1}H spectrum of 6-Me-TEGua (Fig. S6)
5. ^{1}H and ^{13}C NMR spectra of 6-Me-TEG (Figs S7–S8)

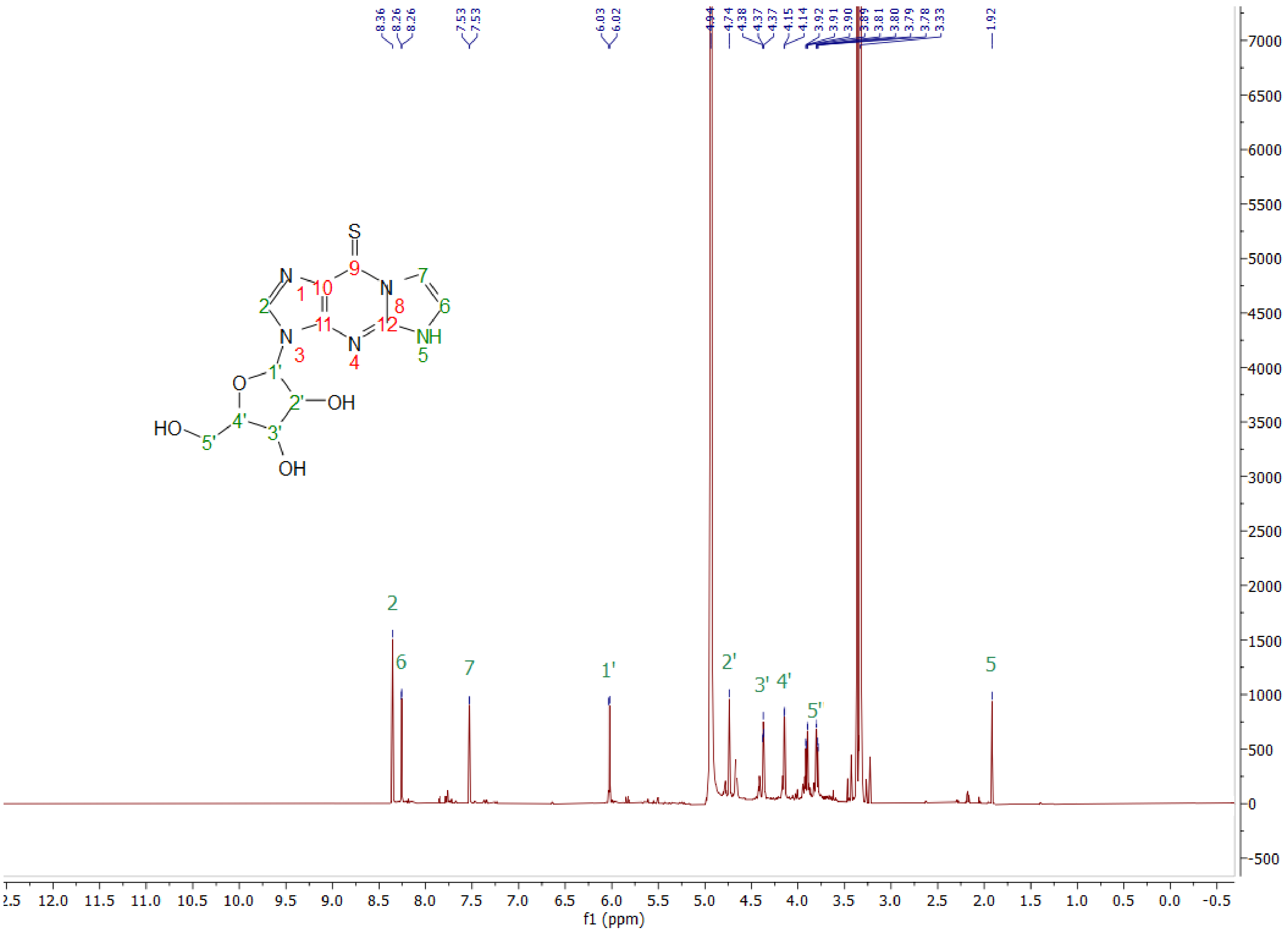

Figure S1. ^{1}H NMR spectrum (MeOD) of TEGuo.

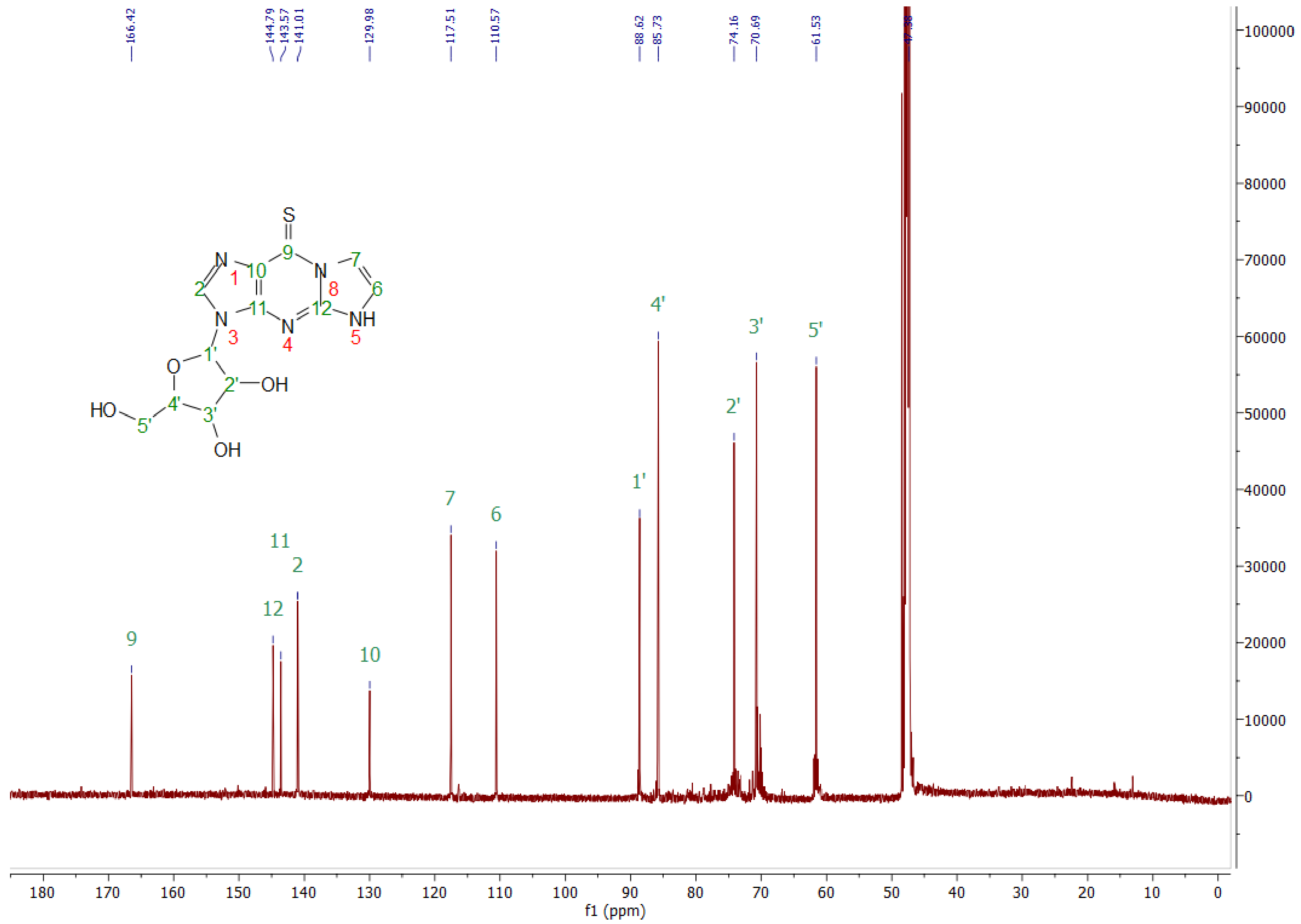

Figure S2. ^{13}C NMR spectrum (MeOD) of TEGuo.

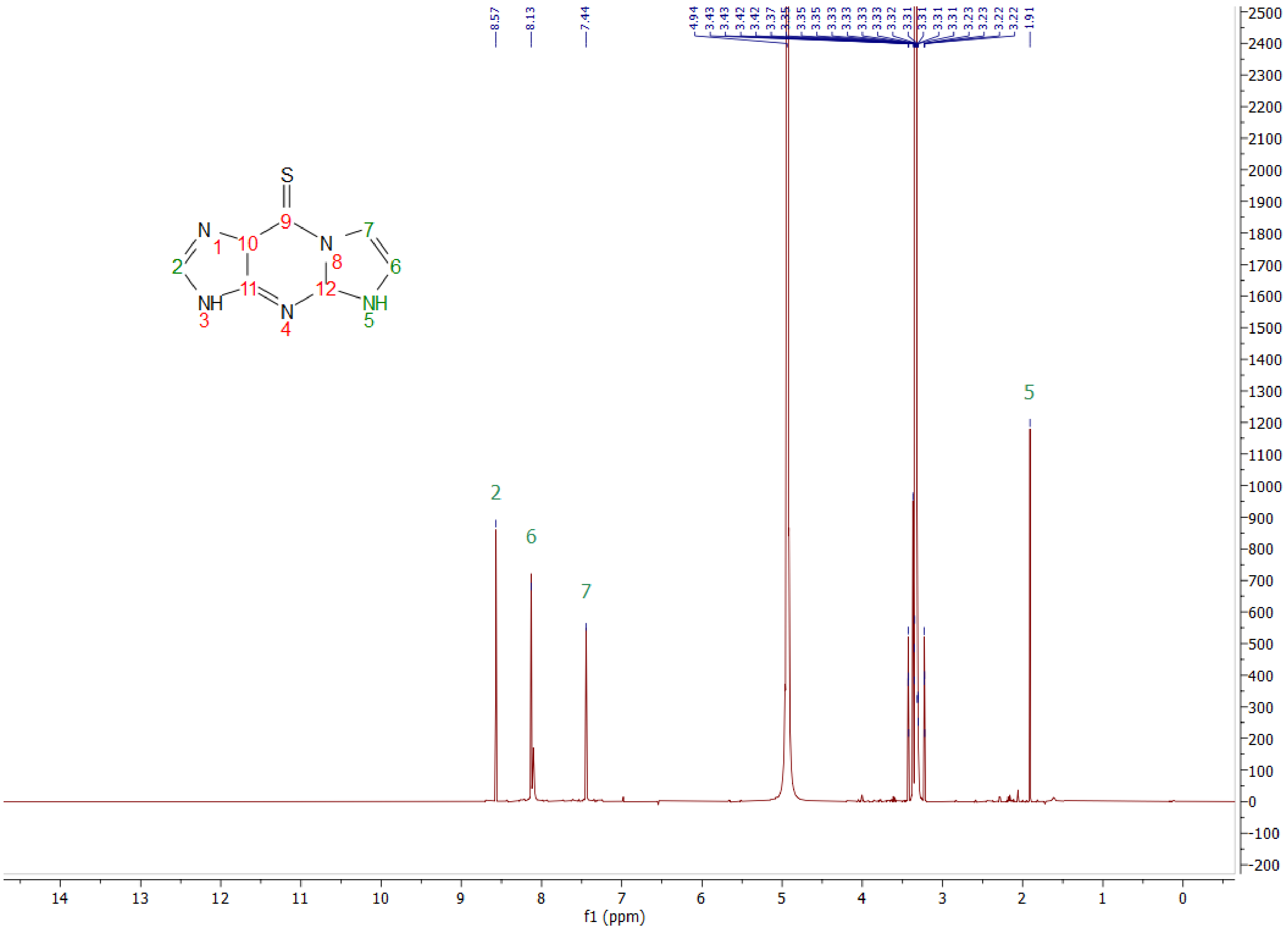

Figure S3. ^{1}H NMR spectrum (MeOD) of TEGua.

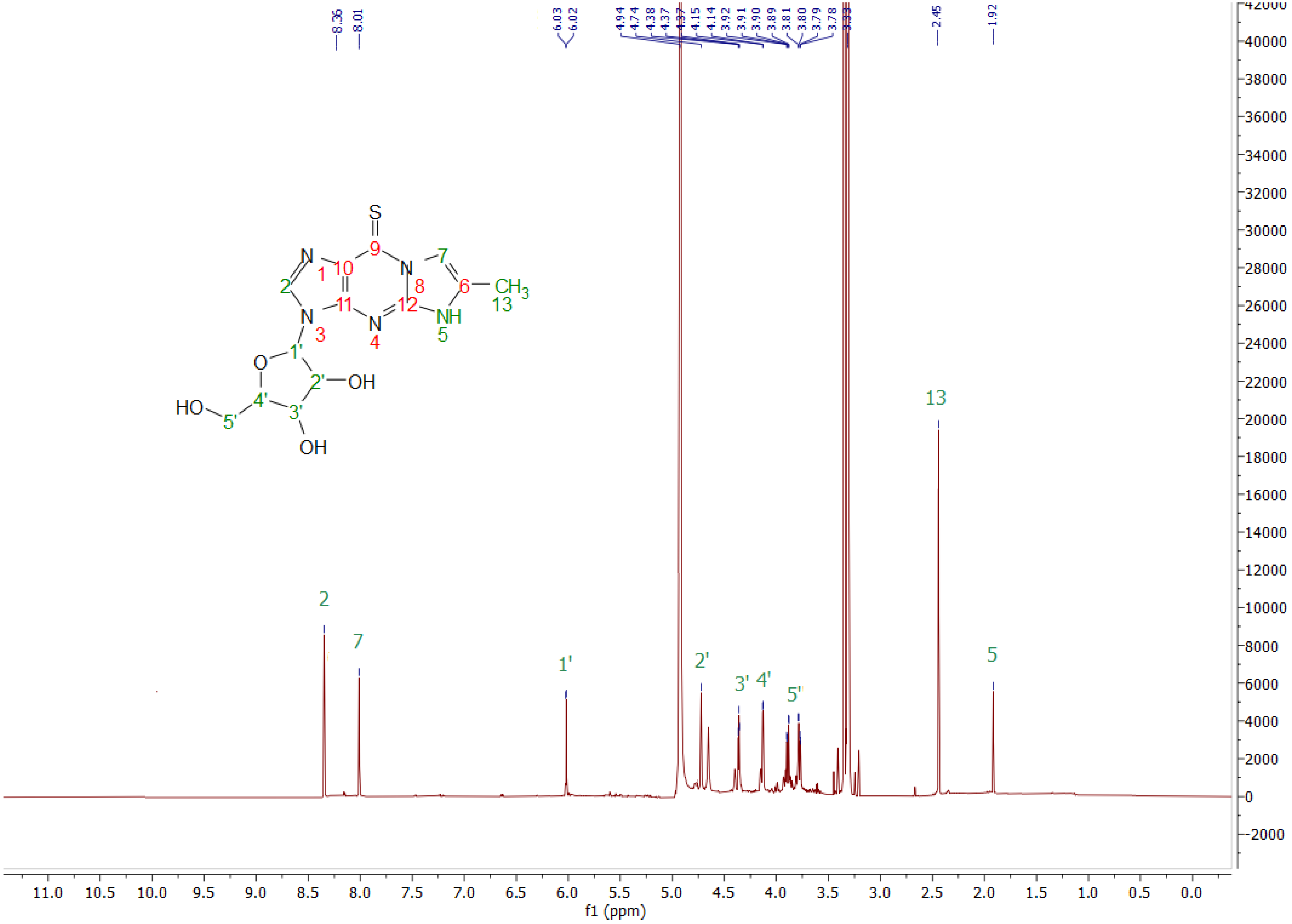

Figure S4. ^{1}H NMR spectrum (MeOD) of 6-Me-TEGuo.

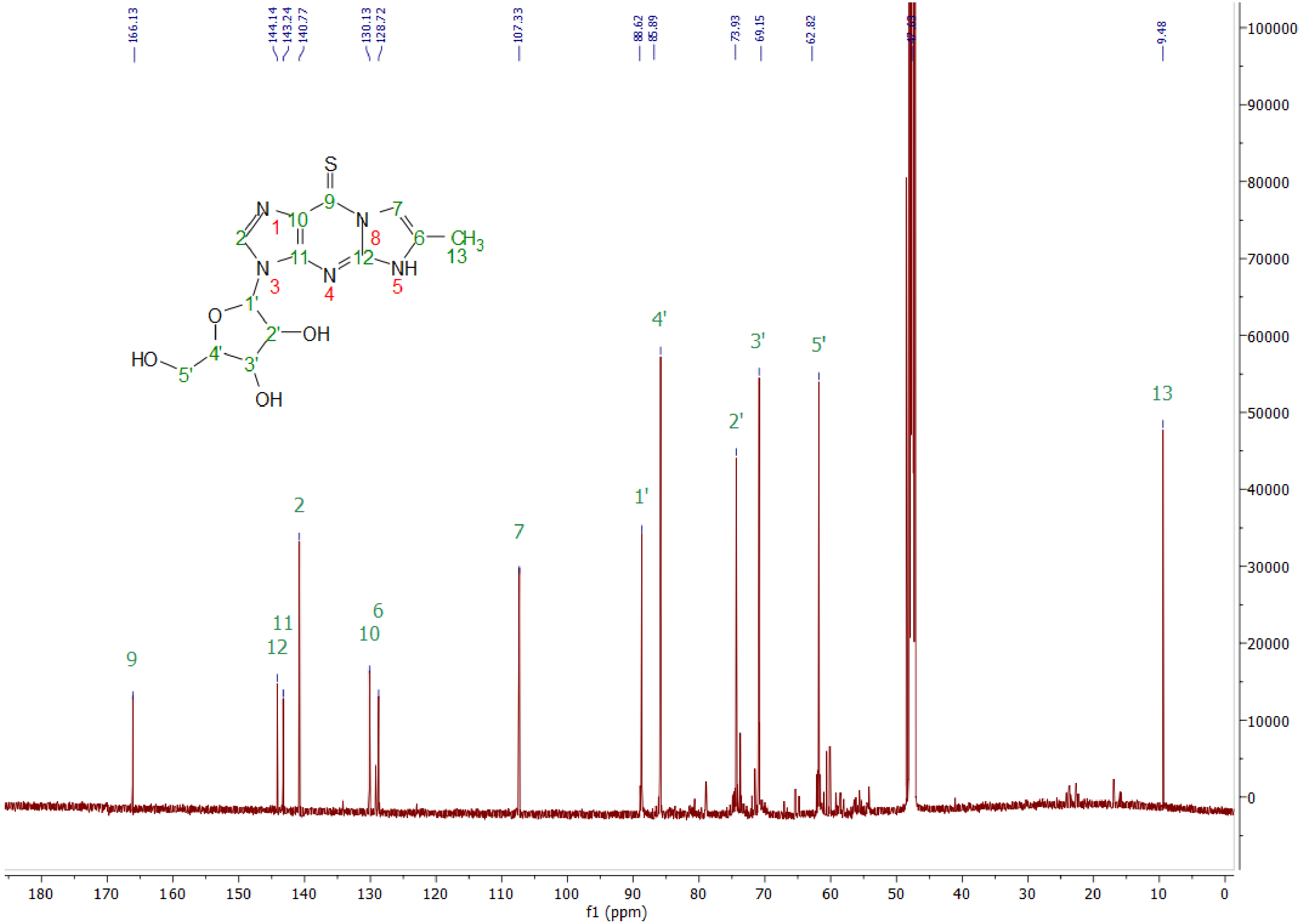

Figure S5. ^{13}C NMR spectrum (MeOD) of 6-Me-TEGuo.

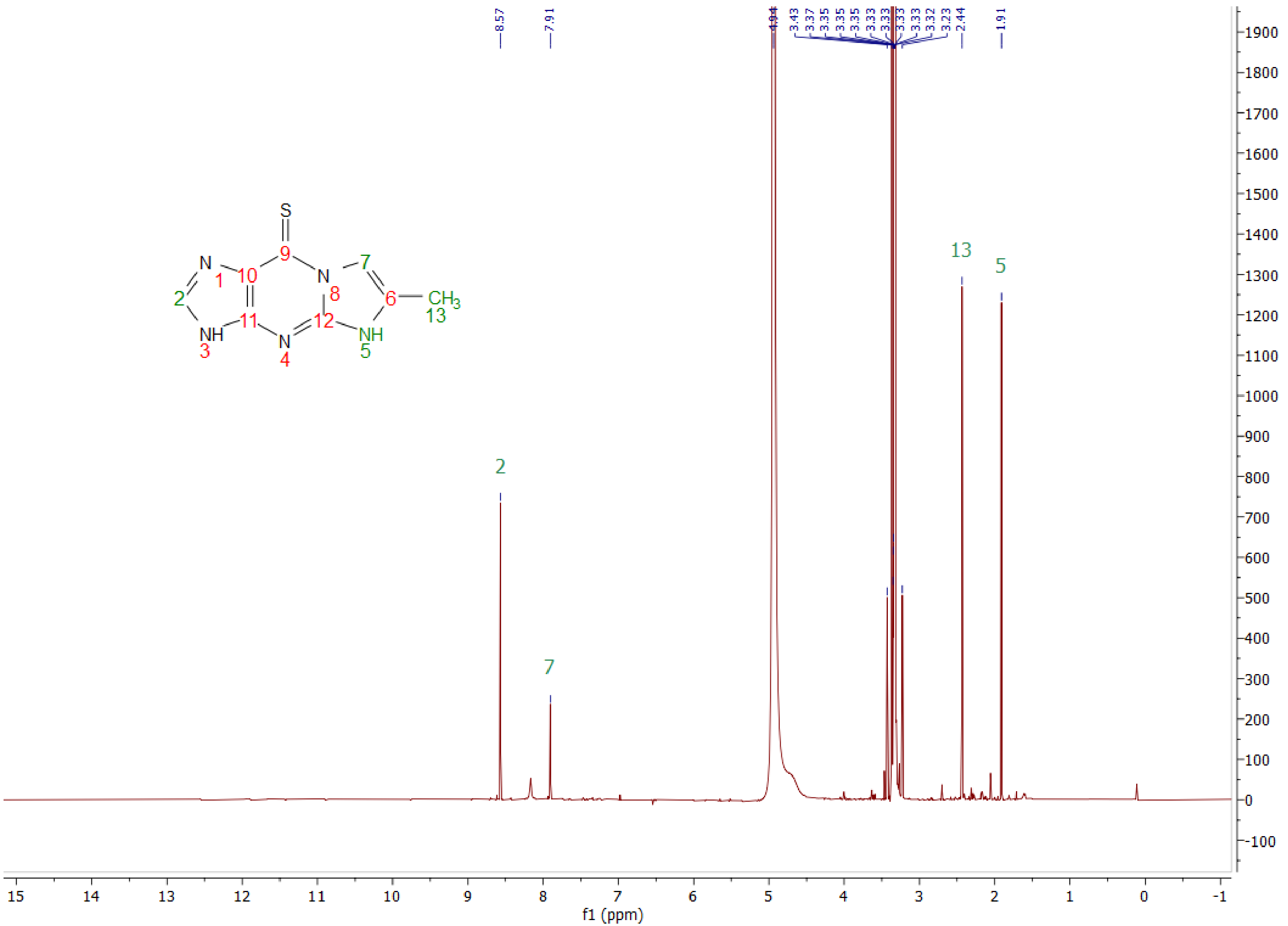

Figure S6. ^{1}H NMR spectrum (MeOD) of 6-Me-TEGua.

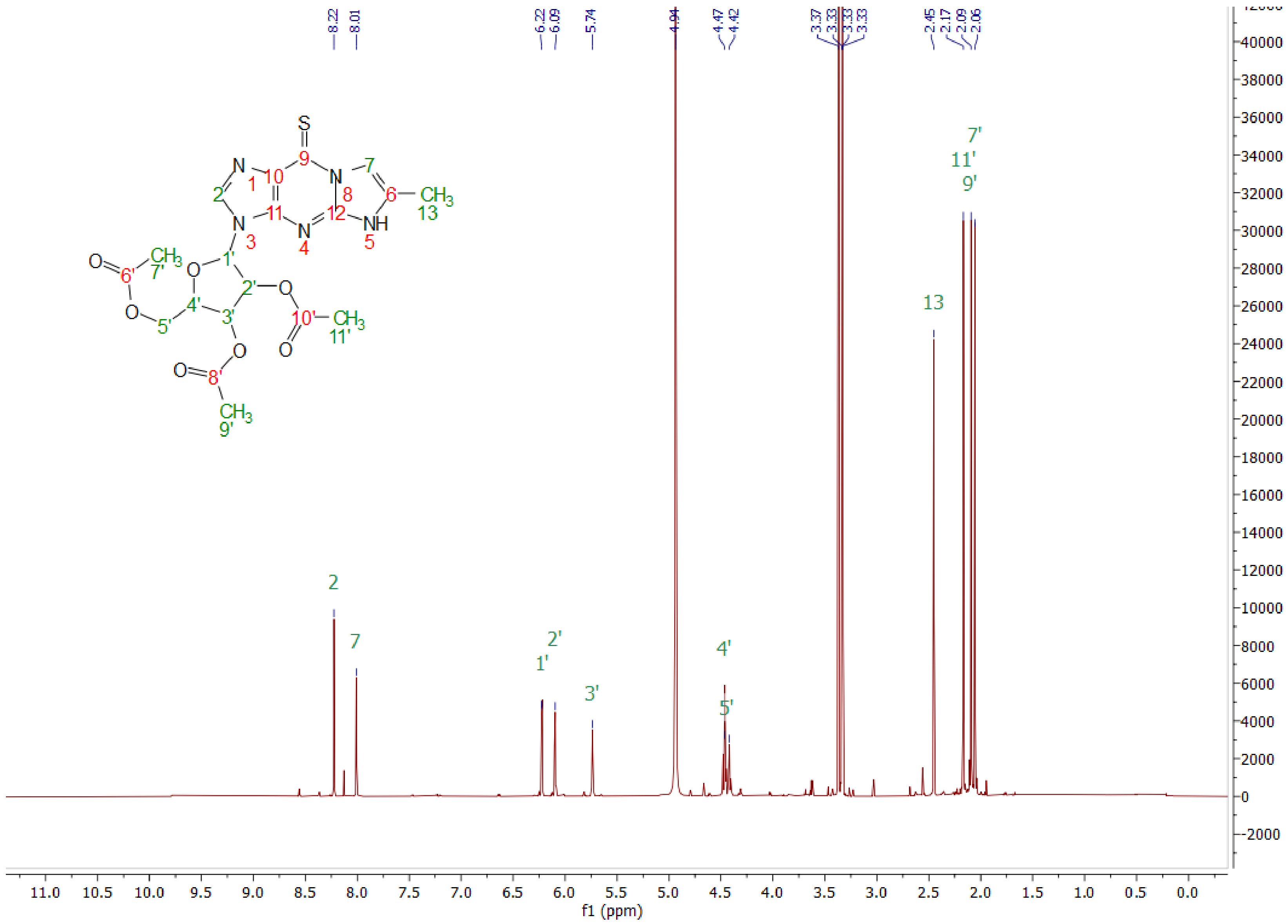

Figure S7. ^{1}H NMR spectrum (MeOD) of 6-Me-TEG.

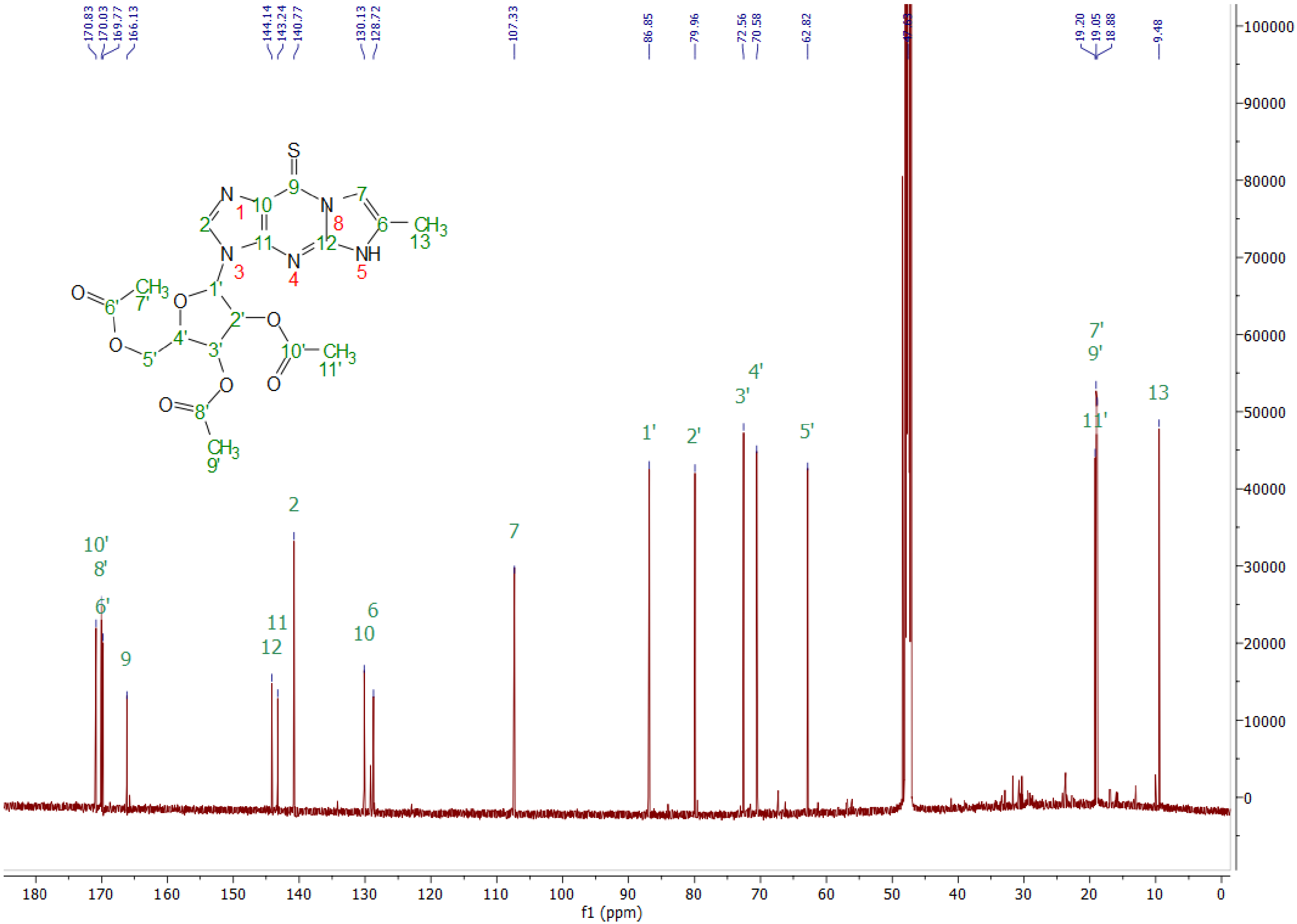

Figure S8. ^{13}C NMR spectrum (MeOD) of 6-Me-TEG.
